# An Antiviral Response Directed by PKR Phosphorylation of the RNA Helicase A

**DOI:** 10.1371/journal.ppat.1000311

**Published:** 2009-02-20

**Authors:** Anthony J. Sadler, Olivier Latchoumanin, David Hawkes, Johnson Mak, Bryan R. G. Williams

**Affiliations:** 1 Centre for Cancer Research, Monash Institute of Medical Research, Monash University, Clayton, Victoria, Australia; 2 The Burnet Institute for Medical Research and Public Health, Melbourne, Victoria, Australia; University of Washington, United States of America

## Abstract

The double-stranded RNA-activated protein kinase R (PKR) is a key regulator of the innate immune response. Activation of PKR during viral infection culminates in phosphorylation of the α subunit of the eukaryotic translation initiation factor 2 (eIF2α) to inhibit protein translation. A broad range of regulatory functions has also been attributed to PKR. However, as few additional PKR substrates have been identified, the mechanisms remain unclear. Here, PKR is shown to interact with an essential RNA helicase, RHA. Moreover, RHA is identified as a substrate for PKR, with phosphorylation perturbing the association of the helicase with double-stranded RNA (dsRNA). Through this mechanism, PKR can modulate transcription, as revealed by its ability to prevent the capacity of RHA to catalyze transactivating response (TAR)–mediated type 1 human immunodeficiency virus (HIV-1) gene regulation. Consequently, HIV-1 virions packaged in cells also expressing the decoy RHA peptides subsequently had enhanced infectivity. The data demonstrate interplay between key components of dsRNA metabolism, both connecting RHA to an important component of innate immunity and delineating an unanticipated role for PKR in RNA metabolism.

## Author Summary

Our manuscript explores the immune response to viral infection by investigating events triggered by the protein kinase PKR. This sentinel kinase is constitutively expressed in all cells as an inactive protein that is subsequently activated by viral RNA produced during an infection. The active kinase perturbs viral replication by phosphorylating protein substrates in the cell. In this manuscript we identify a novel substrate for PKR, an essential helicase, RHA. Viruses usurp this helicase to replicate their own genome. We demonstrate that phosphorylation of RHA by PKR perturbs the ability of the helicase to bind viral RNA. Correspondingly, PKR prevents the capacity of RHA to enhance expression of genetic elements encoded by the human immunodeficiency virus (HIV). Juxtaposed to this, HIV virions packaged within cells that also express protein fragments of RHA, demonstrated to interact with PKR as decoy substrates, have enhanced infectivity. These fragments of RHA occur within a protein domain previously established to bind RNA but increasingly recognized to mediate protein–protein interactions. This supports an emerging role for these protein domains to coordinate the cell's response to pathogen-associated RNA. The findings identify a new cell-signaling pathway important in the response to viral infection.

## Introduction

The primary detection of viral infection is by the host innate immune system, with the recognition of viral double-stranded RNA (dsRNA) a crucial early function. Responses to dsRNA are mediated by several protein receptors that recognize this pathogen-associated molecular pattern (PAMP). Most important of these receptors are the Toll-like receptor-3 (TLR3), two caspase recruitment domain (CARD)-containing helicases, retinoic acid inducible gene-I (RIG-I) and the related IFN inducible helicase-I (IFIH-I), and the protein kinase R (PKR). These dsRNA receptors are spatially separated within the cell to respond to either intra- or extra-cellular dsRNA. Moreover, the outcome of the ensuing antiviral response triggered by each receptor differs between cell compartments [1]. Consequently, a full contingent of pattern recognition receptors is required for immune competence. TLR3 is located on the cell surface or in the endosome compartment, and upon sensing dsRNA recruits the cytoplasmic adaptor Toll/IL-1R (TIR) domain-containing adaptor-inducing IFNβ (TRIF), via shared TIR homologous regions to mediate antiviral responses [2]–[4]. Adaptor signaling leads to IFN regulatory factor (IRF)3 and IRF7 activation and type-I IFN production [5],[6]. RIG-I and IFIH-I are cytoplasmic receptors which sense dsRNA and subsequently transmit a signal via helicase and CARD domains, respectfully. Activated RIG-I/IFIH-I associate with a mitochondrial anchored CARD adaptor, IPS-1 (also called MAVS, Cardif, or VISA), to activate NFκB and IRF3 and induce IFNβ [7]–[10]. Alternatively, dsRNA-binding at the amino terminus of PKR activates the kinase, resulting in the phosphorylation of the α subunit of the eukaryotic translation initiation factor 2 (eIF2α) and inhibition of protein translation within infected cells [11]. In addition, PKR evokes cellular responses by modulating cell-signaling pathways. The mechanisms by which PKR functions as a signaling molecule have not been fully delineated. However, PKR has been shown to mediate the responses to other PAMPs, including bacterial LPS, as well as stress stimuli such as IFNγ, TNFα, mitomycin C, and serum deprivation by inducing degradation of inhibitor κB (IκB), IRF1 expression, indirectly mediating STAT1 phosphorylation, and triggering apoptotic pathways [12],[13]. These broad responses are not reconciled with a narrow mechanism involving translational control through eIF2α. However, few other PKR substrates are known that account for these cellular responses.

PKR has two domains, a C-terminal catalytic domain and an N-terminal regulatory domain. The N-terminus encodes tandem RNA-binding motifs (RBMs). The RBMs not only recognize dsRNA to activate PKR, but also serve as an autoinhibitory domain, as well as mediating dimerization to form the fully active kinase molecule. These observations suggest an additional function for RBMs as protein–protein interaction domains. Support for this comes from other proteins identified to interact with PKR. The protein activator of PKR (PACT) encodes three RBMs, and the structurally similar transactivating response (TAR)-RNA binding protein (TARBP) interacts with PKR to, conversely, inhibit the kinase. That RBMs might mediate interactions between proteins, particularly other RBMs, highlights an emerging concept that has consequence for coordinating the dsRNA response in cells. In this way the RBM might be considered as a signaling domain, analogous to the TIR domain of TLRs or the CARD domain of RIG-I and IFIH-I to mediate homo- or heterotypic protein interactions.

Here, we identify an interaction between PKR and another protein encoding RBMs, the RNA helicase A (RHA). RHA is an essential DEAH-box protein that exhibits both RNA and DNA helicase activity [14]. The association is demonstrated to be via the helicase RBMs. Importantly, biochemical analysis shows RHA is a substrate for PKR, and demonstrates that phosphorylation modulates the helicase association with its nucleic acid substrate. The consequences of these observations are examined in relation to RHA's previously established role in retroviral infection. PKR is shown to mediate transcriptional activity and HIV-1 infectivity via phosphorylation of RHA. These findings identify a novel function for PKR, delineating a new cell signaling pathway to target in anti-HIV-1 therapy, and highlighting a process by which proteins that respond to dsRNA may be coordinated.

## Results

### PKR Associates with RHA In Vivo

**Figure 1:**
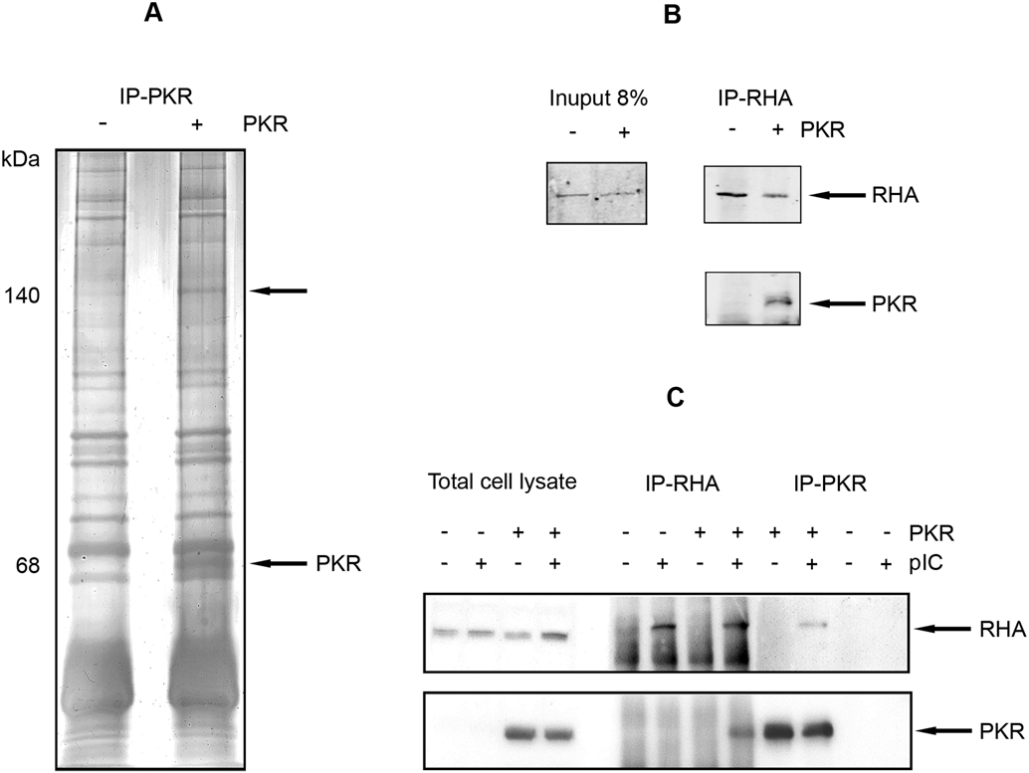
PKR associates with RHA. (A) A silver-stained, SDS-PAGE gel of electrophoretically separated proteins immunoprecipitated with mouse monoclonal anti-PKR (IP-PKR) from *pkr*-null MEFs transformed with a human *pkr* construct either expressed (+) or silenced (−) and stimulated with pIC to activate the kinase. Arrows indicate the 68 kDa PKR and an associated 140 kDa protein. (B) A Western blot of proteins immunoprecipitated using a mouse monoclonal antibody to RHA (IP-RHA) from MEFs isolated form wild-type (+) or *pkr*-null (−) mice. Coimmunoprecipitated proteins are detected with rabbit polyclonal antibodies to RHA (upper panel), or murine PKR (lower panel). The levels of RHA expressed in the different cells were detected in whole cell lysates with the polyclonal anti-RHA antibody (Input). (C) A Western blot of proteins immunoprecipitated using antibodies to either PKR or RHA from *pkr*-null MEFs transformed with human PKR either expressed (+) or silenced (−) and differentially treated with pIC. Coimmunoprecipitated proteins are detected with opposing antibodies for RHA (upper panel) and PKR (lower panel).

To detect proteins interacting with PKR, the kinase was immunoprecipitated from isogenic, *pkr*-null mouse embryonic fibroblasts (MEFs) either silenced or expressing human PKR from the native promoter elements at physiologic levels. The cells were stimulated with the synthetic, dsRNA mimic polyinosinic-polycytidylic acid (pIC) to activate the kinase. A 140 kDa protein band was coimmunoprecipitated with a monoclonal anti-PKR antibody (Figure 1A). Mass spectrometric analysis of this protein identified 11 different peptide sequences that matched the amino acid sequence of murine RHA (Table 1).

**Table 1:** Mass spectrophotometry sequencing of the PKR-associated protein

Peptide Sequence	Matched RHA Residues
NFLYAWCGK	6–14
DAQSNAAR	56–63
DFVNYLVR	64–71
AENNSEVGASGYGVPGPTWDR	121–141
GANLK	142–146
DYYSRK	147–152
LIQYFQK	185–191
EKIQGEYK	192–199
YTQVGPDHNR	200–209
SFIAEMTIYIK	210–220
LAAQSCALSLVR	237–248

Immunoprecipitation and Western blot analysis were conducted to verify this protein association. The protein interaction was confirmed in MEFs isolated from wild-type and *pkr* null mice by performing the reciprocal immunoprecipitation, using a anti-RHA monoclonal antibody, then detecting coimmunoprecipited PKR (Figure 1B). This experiment was repeated in the rescued *pkr* null MEFs expressing the human kinase, with and without stimulation of the cells with pIC to activate PKR. Coimmunoprecipitation of PKR and RHA only occurred with pIC treatment, demonstrating that the protein interaction is dependent upon activation of the kinase (Figure 1C). Notably, pIC appears to modulate the conformation of RHA as the protein is not immunoprecipitated by its own antibody from untreated cell lysates. This supports a previous suggestion that the constitutively expressed helicase is maintained in an inactive conformation, likely via the protein's RBM, under basal conditions [15].

### PKR Associates Directly with the N-Terminus of RHA

**Figure 2:**
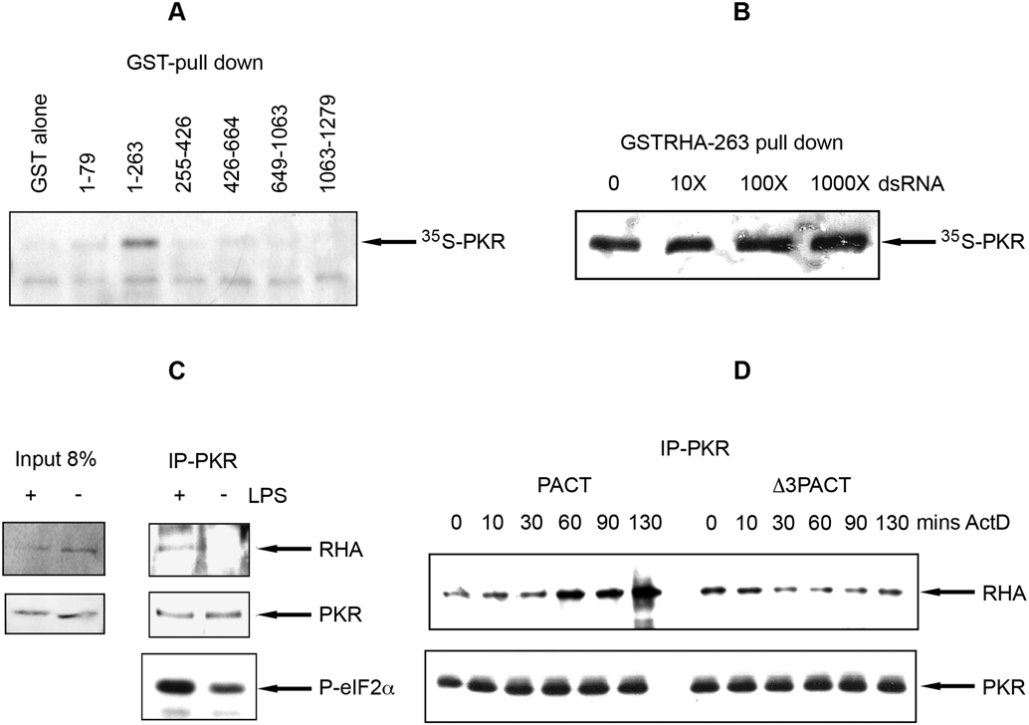
Activated PKR directly associates with the N-terminus of RHA. (A) An autoradiograph of ^35^S-labeled PKR, pulled down with GSTRHA fusion proteins and GST alone. Amino acid end points for the RHA construct are indicated over each lane. (B) An autoradiograph of ^35^S-labeled PKR pulled down with a GSTRHA fusion protein encoding amino acid residues 1 to 263 in a competitive reaction with 0, 10, 100, or 1000 fold excess of a 16 nucleotide dsRNA. (C) A Western blot of proteins immunoprecipitated with a monoclonal antibody to PKR (IP-PKR) from lysates of the human monocyte THP-I cell line, differentially treated (+/−) with LPS to activate the kinase. Proteins are detected with rabbit anti-RHA (top panel) or mouse anti-PKR (middle panel). Activation of PKR was confirmed by detection with a rabbit anti-phosphoS51-eIF2α (P-eIF2α; bottom panel). (D) A Western blot of proteins immunoprecipitated with a monoclonal antibody to PKR (IP-PKR) from lysates of HEK293T transfected with HA-tagged RHA and PACT or the deletion construct Δ3PACT and temporally treated with actinomycin-D (mins ActD) to activate PKR. Proteins were detected with a rabbit anti-HA (RHA; top panel) and mouse anti-PKR antibody (bottom panel).

In vitro binding studies were conducted to map the association between PKR and RHA. Six different glutathione-S-transferase (GST) fusion constructs, together spanning the full helicase, were used in a GST-pull down experiment to determine which region of RHA associated with PKR. This experiment showed PKR associated exclusively with the first 263 amino acids of RHA that encodes its two RBMs (Figure 2A). Since the first RBM, encoded between amino acids 1 to 79, did not associate with PKR, it appears that the second RBM is the interacting region, or both RBMs are required. Efforts to map the region of PKR that interacts with RHA were inconclusive. Binding assays with the isolated RBM or kinase domains of PKR showed the N-terminal RBMs interacted specifically with RHA. However, the truncated C-terminus of PKR bound non-specifically to the control (beads only) in the assay conditions. Subsequent analysis suggests the RBM of RHA interacts with both C- and N-terminal domains of PKR (see below).

RHA encodes several domains that bind dsRNA, located in the helicase domain, the C-terminal RGG box, as well as the two RBMs at the N-terminus, that did not associate with PKR. This implies that the PKR-RHA interaction is direct and not through mutual association with dsRNA. Moreover, it has been established that PKR dissociates from dsRNA upon activation, autophosphorylation and dimerization [16]. Consequently, an indirect association between PKR and RHA, bridged by dsRNA, is unlikely. However, to unequivocally establish that the two proteins interact directly, several experiments were conducted.

First the ability of a 16 bp dsRNA molecule to block the association between in vitro synthesized PKR and the N-terminal 263 amino acid GST-fusion construct of RHA was measured. This short dsRNA, although able to bind to a single RBM, is not long enough to interact with two RBMs from separate proteins [17]. The results (Figure 2B) showed that the 16 bp dsRNA did not perturb the association between PKR and RHA, even when present at considerable molar excess. Next, alternative cell treatments that did not use pIC to activate PKR were evaluated and coimmunoprecipitations performed. Accordingly, treatment of the human monocytic cell line, THP-1, with LPS, demonstrated to activate PKR [18], induces the association between the kinase and RHA (Figure 2C). Finally, triggering of PKR with its protein activator PACT [19] effected association between PKR and RHA. HEK293T cells were cotransfected with expression constructs for RHA and wild-type PACT or a mutant (ΔPACT) that does not activate PKR and treated with actinomycin-D to stimulate PACT, then PKR was immunoprecipitated. Figure 2D shows the wild-type and not the mutant PACT increased the association between PKR and the helicase. Together these data demonstrate that PKR interacts directly with the N-terminal region of RHA, and that this interaction is dependent upon activation of the kinase.

### RHA Is a Substrate for PKR

**Figure 3:**
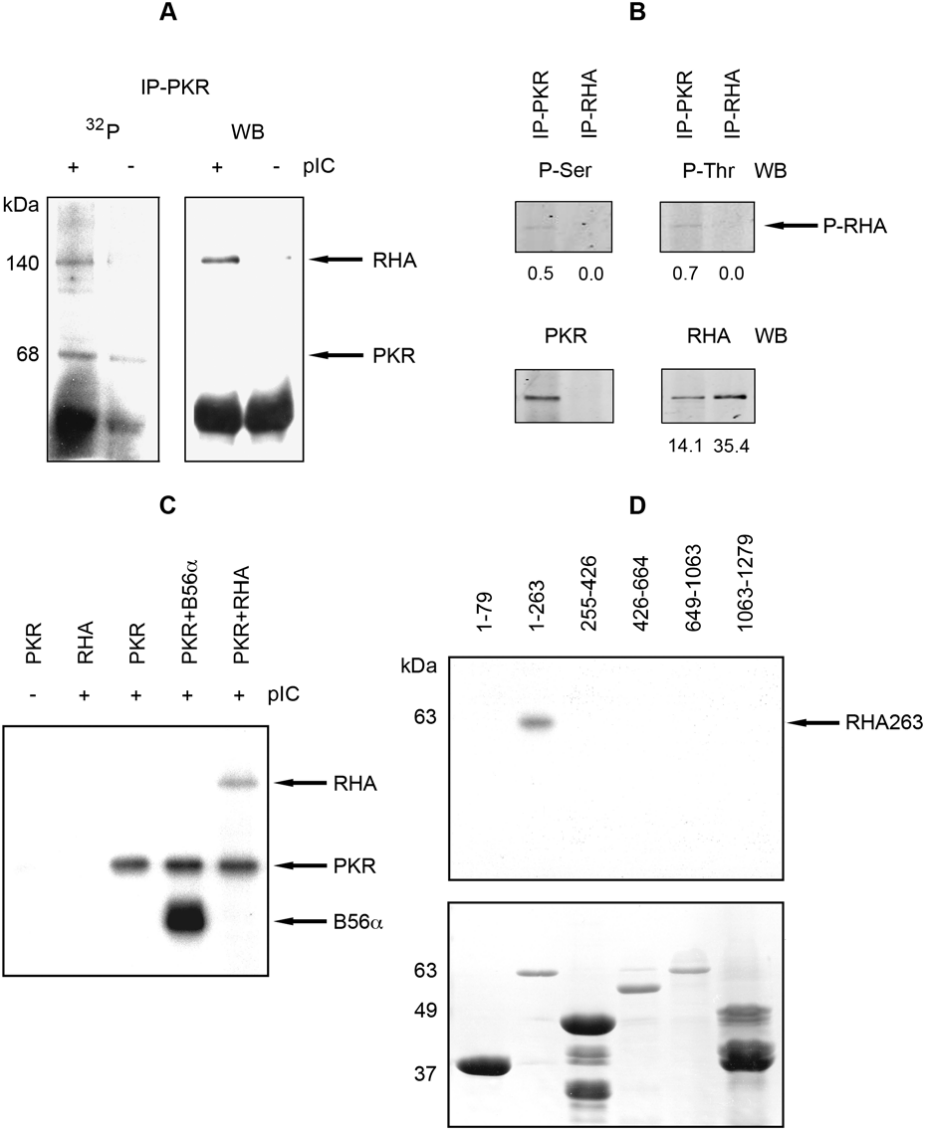
RHA is a substrate for PKR. (A) An autoradiograph (32P; on the left) and Western blot (WB; on the right) showing electrophoretically separated proteins labeled with ^32^P by PKR in a kinase assay, or probed with a rabbit anti-RHA antibody, respectively. Proteins were immunoprecipitated with mouse anti-PKR (IP-PKR) from MEFs expressing human PKR either untreated (−) or treated (+) with pIC. (B) A Western blot showing electrophoretically separated proteins immunoprecipitated from HeLa cells with anti-PKR (IP-PKR) or anti-RHA (IP-RHA) antibodies, then probed with anti-phosphoserine (P-Ser) and anti-phosphothreonine (P-Thr). Arrows indicate phosphorylated RHA (P-RHA). Blots were stripped and re-probed with anti-RHA, or anti-PKR antibodies. Primary antibodies were detected using fluorescent-labeled secondary antibodies. The intensity of the immune-positive bands from the blot was quantitated, and these values are given at the bottom of each panel as relative arbitrary units. (C) An autoradiograph of electrophoretically separated proteins labeled with ^32^P by PKR in an in vitro kinase assay. Arrows indicate autophosphorylated PKR and phosphorylated RHA, and, as a control, the previously established PKR substrate B56α. PKR activity is induced by treatment with pIC, indicated at the top of the figure (−/+). (D) An autoradiograph of GSTRHA peptides ^32^P-labeled by PKR in a kinase assay and electrophoretically separated by SDS-PAGE gel (upper panel). Amino acid end points for each RHA construct are indicated over each lane. The arrow indicates the phosphorylated RHA peptide (RHA263). The lower panel shows the Coomassie-stained, SDS-PAGE gel assayed above.

To investigate the possible consequences of this interaction, we tested whether RHA is a substrate for PKR-mediated phosphorylation. An in vitro kinase assay was performed with proteins coimmunoprecipitated from MEFs using a monoclonal antibody against PKR. The results (Figure 3A) show RHA bound to PKR at physiological ratios was phosphorylated by the associated PKR in a subsequent kinase assay. To measure phosphorylation in vivo RHA was directly immunoprecipitated with antibodies to RHA, or coimmunoprecipitated with an anti-PKR antibody from HeLa cells treated with pIC. The resulting immune complexes were probed by western blot for phosphorylated residues using anti-phosphoserine and anti-phosphothreonine antibodies. Figure 3B shows only the RHA in complex with PKR had detectable phosphorylated serine and threonine residues. Detection of phosphorylated RHA specifically associated with the kinase, strongly suggesting direct phosphorylation of RHA by PKR. The antibody used to immunoprecipitate RHA in this experiment (ab2627 from Abcam) was raised against a synthetic peptide derived from within residues 100 to 200 of human RHA. This is within the region of RHA demonstrated to associate with PKR (between residues 80 to 263, Figure 2A). As this antibody and PKR interacts with the same region of RHA mutual association is excluded. Consequently, PKR is not coimmunoprecipitated with this anti-RHA antibody (Figure 3B). This confirms the GST-pull down experiments and further narrows the region mediating the interaction between the RHA and PKR.

Phosphorylation of RHA by PKR was confirmed in an in vitro kinase assay, using purified recombinant PKR and RHA (Figure 3C). To examine the possible functional consequences of phosphorylation by PKR we mapped the region of RHA that is modified. Accordingly, an in vitro kinase assay was conducted with truncated GST-fusion constructs of RHA and recombinant PKR. Figure 3D demonstrates that RHA is phosphorylated within the 263 amino acid region previously demonstrated to interact with PKR. Hence, the RBM of RHA must interact with the catalytic kinase domain of PKR. This is consistent with previous evidence showing other RBMs, for instance from PACT, are phosphorylated by PKR [20],[21].

### PKR Perturbs the Association of RHA with RNA

**Figure 4:**
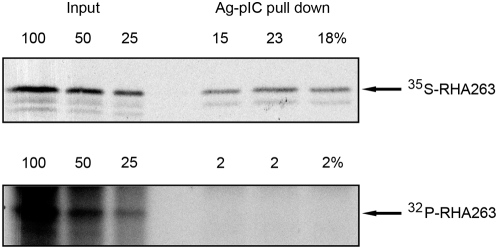
PKR phosphorylation perturbs the association of RHA with RNA. A phosphor image of the recovery of total ^35^S-labeled RHA peptide encoding amino acid residues 1 to 263 (top panel) compared to ^32^P-labeled peptide phosphorylated by PKR in vitro (bottom panel) pulled down with pIC bound to agarose beads (Ag-pIC) and electrophoretically separated. The recovery of three replicated radioactively labeled peptides was quantitated as a percentage of the input extract (Input).

As the N-terminal 263 amino acid region of RHA regulates the association with dsRNA, it seemed evident that addition of a negatively charged phosphate group to this region would perturb RHA's interaction with dsRNA. To test this hypothesis we measured the relative affinity of the phosphorylated or unphosphorylated RHA peptide for pIC. The 263 amino acid RHA peptide was either taken directly from an in vitro synthesis reaction, or subsequently phosphorylated by PKR in an in vitro kinase assay following synthesis. Approximately 19% of the total ^35^S-labeled RHA peptide was recovered in a pIC pull down. In contrast, none of the phosphorylated RHA peptide, evidenced as a ^32^P-labeled product, bound pIC (Figure 4). Consequently, phosphorylation of the RHA peptide by PKR inhibited pIC binding. Given that phosphorylation perturbs RHA's association with its nucleic acid substrate, we would expected PKR should have a profound effect upon the function of the helicase in vivo.

### PKR Inhibits RHA Function

**Figure 5:**
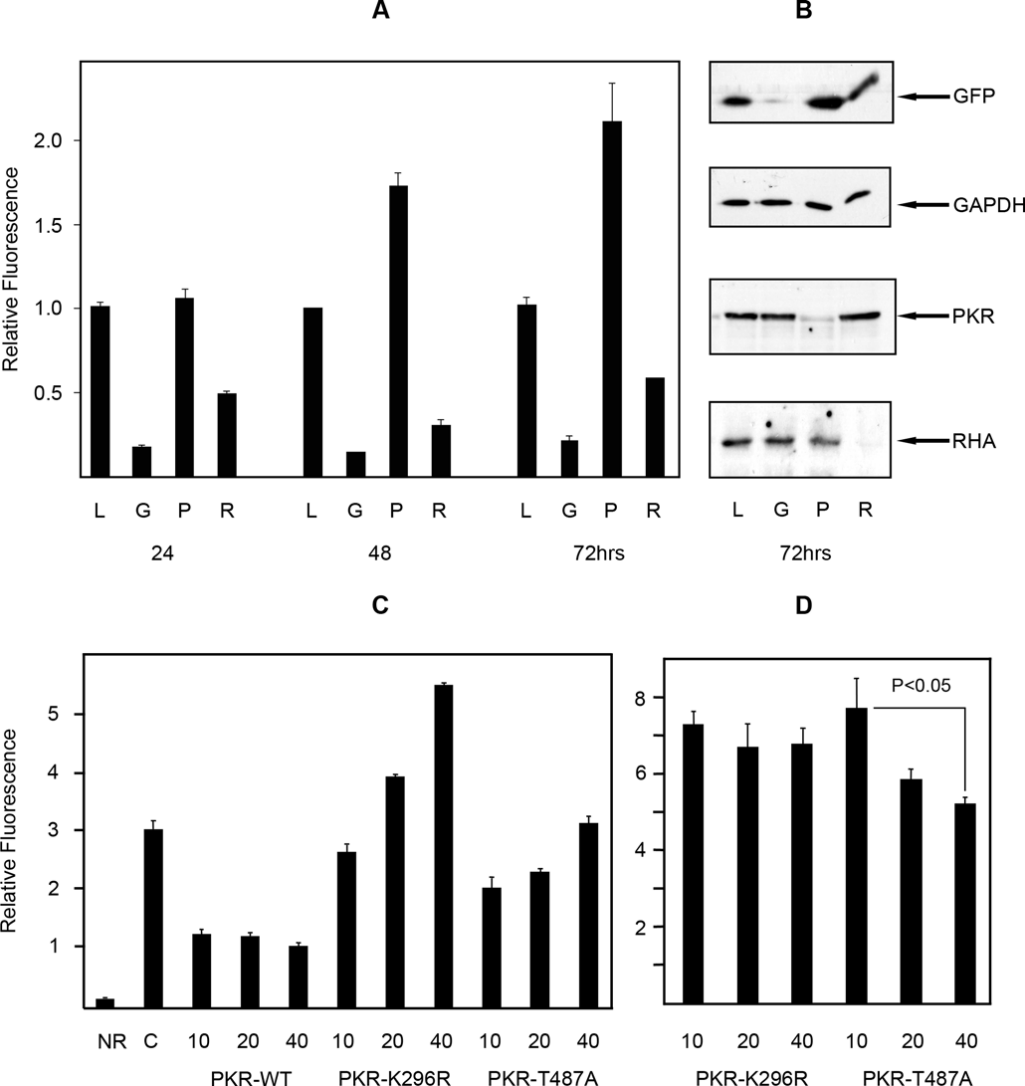
PKR inhibits RHA-mediated transactivation of HIV-1 LTR. (A) A bar graph representing the relative fluorescence levels in transiently transfected HEK293T cells using a HIV-1 LTR-EGFP reporter and siRNAs against *lamin a/c* (L), *gfp* (G), *pkr* (P), or *rha* (R), to measure the effect of PKR on RHA-mediated LTR-transactivation. Cell lystates were collected and assayed at three time points (24, 48, and 72 hours). Each value is the average of three independent measures, and the error bars represent the standard deviations. Fluorescence measures were corrected to a constitutive βactin-renilla reporter, then normalized to the *Lamin a/c* siRNA treatment. (B) Western blots of electrophoretically separated proteins, from HEK293T cells treated as in (A). Proteins were detected with antibodies to GFP, GAPDH, PKR, and RHA to show intended depression of targeted proteins and PKR-dependent control of RHA-mediated LTR-transactivation. (C) A bar graph representing the total relative fluorescence levels in HEK293T cells alone (NR), or transiently transfected with a HIV-1 LTR-EGFP reporter (C), and with wild-type PKR (PKR-WT), a kinase inactive mutant (PKR-K296R) or a mutant that does not regulate translation via eIF2α (PKR-T487A), at three concentrations (10, 20, and 40 ng/well) to show kinase activity is required to regulate RHA-mediated induction of the HIV-1-LTR promoter sequence. Fluorescence levels are not normalized against a constitutive βactin reporter. Each value is the average of three independent measures, and the error bars represent the standard deviations. (D) The relative fluorescence levels in HEK293T cells transfected with the HIV-1 LTR-EGFP reporter and a kinase inactive mutant (PKR-K296R), or a translational control mutant (PKR-T487A) from (C) normalized against a constitutive βactin reporter to show specific, dose-dependent PKR-regulated inhibition of the RHA activity. A t-test was performed on the data to establish the statistical significance of the observed PKR-mediated reduction in the LTR reporter.

Our data supports a model in which PKR regulates RHA by phosphorylating its RBD thereby decreasing its affinity for RNA. To determine the in vivo consequence of such regulation, we investigated PKR's effect on the reported ability of RHA to regulate transactivation of the HIV-1 LTR [22]. Accordingly, transcription of an LTR-EGFP reporter construct was measured in HEK293T cells in which PKR was depleted by RNA interference (RNAi). Additional control small interfering RNAs (siRNAs) against RHA, EGFP, and as an alternative target Lamin A/C, were cotransfected with the reporter construct to gauge the RHA dependence of transactivation, the efficacy of RNAi, and to account for non-specific effects of RNAi, respectively. Since the HIV-1 LTR RNA has been reported to bind and activate PKR, no further activating stimulus was used [23]. As depletion of PKR can increase gene expression by reduced phosphorylation of the eIF2α translation factor, we delineated specific regulation of LTR-transactivation by normalizing reporter protein levels to an internal constitutive Renilla reporter. Western blot analysis confirmed the specific release of the reporter gene (GFP) relative to the constitutively expressed GAPDH, and verified appropriate targeting of each siRNA against PKR, RHA, and as a control GFP (Figure 5B). The control siRNA to Lamin A/C did not affect reporter protein levels and is not shown. Depletion of RHA by siRNA confirms the role of the helicase in LTR-regulated gene expression (Figure 5A). Significantly, depletion of PKR increased EGFP expression. The timing of transcriptional release, beginning at 48 hours, conforms to the anticipated kinetics of the removal of PKR from the cell, as the kinase has an approximate half-life of 48 hours (B. R. G. Williams, unpublished results).

This effect of PKR on the LTR reporter system was further tested using three PKR constructs with different catalytic activity. The requirement for kinase activity for PKR control of RHA-mediated LTR expression was assessed by comparing the relative affect of wild-type PKR, and two mutant PKR proteins either; catalytically active but modified to preclude eIF2α regulation by substitution of the threonine residue to an alanine at position 487 (T487A) [24], or a kinase dead construct modified by substitution of a lysine residue to a arginine at position 296 (K296R). Expression of wild-type PKR reduced RHA-dependent transcription of the LTR-EGFP reporter. Conversely, expression of the catalytically inactive PKR-K296R promotes RHA-dependent transcription of the reporter (Figure 5C). This construct (K296R) dimerizes with endogenous PKR, so acts as a dominant negative to directly inhibit PKR's regulation of RHA as well as general protein translation, via wild-type PKR phosphorylation of eIF2α. The relative contribution of these two mechanisms was explored by expressing the mutant PKR-T487A that mediates association with eIF2α. This construct is catalytically active, so will phosphorylate RHA, but is incapable of regulating translation. Accordingly, expression of the PKR-T487A showed an intermediate affect on the LTR-driven reporter, reflecting direct inhibition of RHA-mediated induction of the reporter without the wild-type PKR-mediated regulation of global protein translation (Figure 5C). The relative contribution of PKR's direct regulation of RHA juxtaposed to indirect effect upon translation, demonstrated with either PKR mutation (K296R or T487A), is made more clear when the RHA-dependent transcription of the HIV-1 LTR reporter (EGFP) is normalizing against a constitutive reporter (Renilla luciferase). This normalization shows the catalytically inactive PKR-K296R does not affect RHA-mediated transactivation of the HIV-1 LTR, while the catalytically active PKR-T487A construct reduces transactivation of the HIV-1 LTR. This data shows that PKR negatively regulates RHA transactivation of the retroviral reporter gene by direct phosphorylation control of RHA.

### The RHA RBM Increases HIV-1 Infectivity

**Figure 6:**
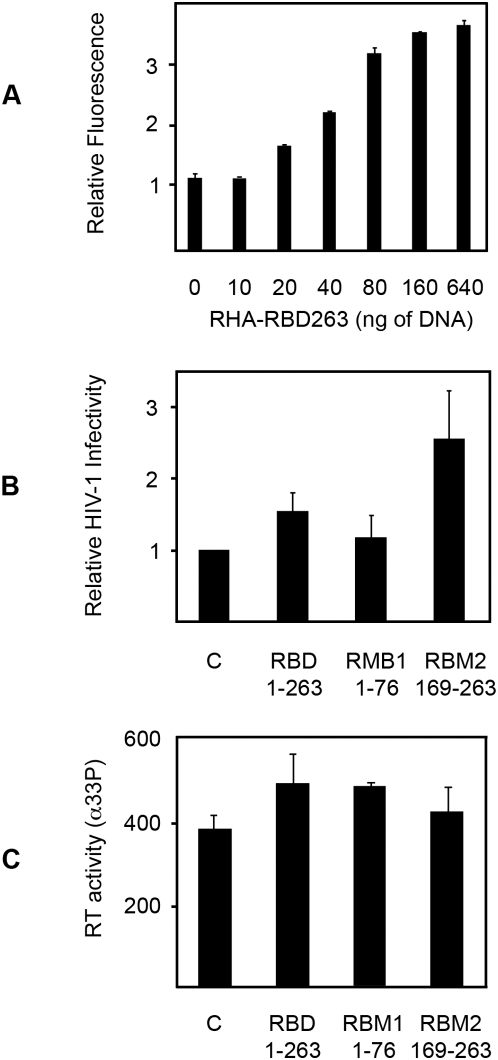
The RBD of RHA enhances HIV-1 infectivity. (A) The graph shows RHA-regulated, LTR-expression is increased in HEK293T cells in proportion to increasing amounts of the N-terminal RHA RBM. Values are normalized to equivalent levels of an empty control plasmid. Error bars represent standard deviations. (B) The effect of different RHA constructs on HIV-1 infectivity was assessed using recombinant virus in the T-cell line MT-2. Values were normalized to HIV-1 produced with an empty control vector. Each value is the average of three independent experiments with replicates. The error bars represent the standard deviations. (C) A bar graph representing the relative replication capacity of HIV-1 produced with different RHA or control constructs (RBD, RBM1, and RBM2). Viral replication is measured by assaying reverse transcriptase activity. The values shown are an average of four replicates. Error bars show the standard deviation.

The preceding data predicts over expression of the RBD of RHA would perturb PKR function by acting as a decoy substrate. This prediction is supported by reporter assays in HEK293T cells that show RHA-regulated LTR expression increases with increasing amounts of the RHA RBD (Figure 6A). This rescue effect of RHA's RBD in the reporter assays should extend to full viral infection. To test this, we measured the capacity of constructs that encoded RHA's RBD, and two truncated constructs, of each separate RBM within this domain, to enhance HIV-1 infection in the peripheral blood mononuclear cell line MT-2. As a previous report had demonstrated that RHA becomes incorporated into the HIV-1 virion during replication [25], infectious virus was produced in cells co-expressing the three RHA peptides (RBD, RBM1, and RBM2, encoding residues 1-263, 1-76, and 169-263, respectively), and the virions produced were titrated onto the mononuclear cells. In keeping with the reporter assays, expression of the RBD significantly increased HIV-1 infectivity. Notably, a truncation construct from the first RBM (RBM1) that did not associate with, and was not phosphorylated by, PKR (Figures 2A and 3D), did not alter HIV-1 infectivity. In contrast, the construct encoding the second RBM (RBM2), predicted to be the substrate for PKR, did enhance viral infectivity. In fact, this peptide was more potent than the domain that encompassed both motifs (Figure 6B). As these truncated constructs have no helicase activity and lack other domains demonstrated to enhance retroviral replication, increased virus infectivity is presumed to be due to the demonstrated association and inhibition of PKR. However, an alternative mechanism is conceivable whereby RHA's RBD might recruit other cellular factors to enhance viral replication. This was assessed by measuring the activity of reverse transcriptase in infections with HIV-1 produced with the control plasmid or each of the RHA constructs. Importantly, the RHA peptides did not increase viral replication, as measured by the activity of the viral reverse transcriptase enzyme (Figure 6C). These experiments validate the preceding data in a cell infection system, substantiating a consequence of the interaction between PKR and RHA for the cell's innate immune response to HIV-1 infection.

## Discussion

The innate immune response is the primary shield against microbial infection and directs the subsequent adaptive response. The protein kinase PKR was identified some 30 years ago as a sentinel kinase that is constitutively expressed in all cells as a monomer that subsequently dimerizes to form the active enzyme. We show here that RHA is a novel substrate for PKR and explore points of significance that arise from the finding.

PKR's interaction with RHA identifies a novel mechanism by which the previously established translational regulator can also modulate transcription. This function of PKR identifies an antiviral pathway that represents a plausible target for treatment of established retroviral infections. Consistent with this antiviral mechanism, RHA is positively associated with viral replication. RHA transactivates the Bovine Viral Diarrhea virus by binding to the terminal nontranslated regions of the viral RNA genome [26]. RHA also positively regulates expression of the HIV-1 transactivation response region [27] (and in this study). In addition, RHA mediates release of retroviral transcripts from the splicesome and transports the RNA from the nucleus. Correspondingly, the helicase has been shown to associate with the constitutive transport element (CTE) of type D-retroviruses and Rev Response elements of HIV-1 and to associate with cellular mRNA export receptors TAP, SAM68, and HAP95 [28],[29]. Of particular relevance to this study, RHA also associates with the HIV-1 gag protein and becomes incorporated into the HIV-1 particle [25]. Our data demonstrates that coexpression of HIV-1 provirus with RHA peptides that are substrates for PKR subsequently enhances viral infectivity. Significantly, the truncated RHA peptides do not encode any helicase activity and are therefore incapable of transactivating the HIV-1 LTR sequences. Appropriately, no benefit to virus replication was observed by co-expressing RHA peptides. We contend the observed increased infectivity, without increased replication, is due to inhibition of the ensuing antiviral response mediated by PKR, through the interaction between RHAs second RBM and PKR. Therefore an additional function of RHA possibly exploited by HIV-1 is to dampen the primary host immune response. Such a role adds weight to the previous observed incorporation of RHA into the virion.

RHA was initially identified as a homolog of the *Drosophila melanogaster maleless* gene that regulates chromosomal dosage compensation, a function essential for survival of male larvae [30]. Deletion of *rha-1* in *Caenorhabditis elegans* indicates that the helicase controls germ cell proliferation and development [31]. Embryonic lethality of *rha* null mice at day 11 of gestation shows that the helicase is also essential for development in mammals [32]. Several lines of evidence suggest that RHA may also have a role in the immune response. The helicase appears as an auto-antigen in the auto-immune disease systemic lupus erythematosus [33]. In addition, RHA associates with the transcription cofactor and histone acetyltransferase CBP, and the transcription factor NFκB, both potent factors in immune responses [34],[35]. Furthermore, the *rha* gene promoter contains regulatory elements that control induction of this constitutively expressed protein during cellular immune responses, including an Interferon Stimulatory Response Element. Interestingly, immunohistochemistry of IFNα-treated cells shows accumulation of the helicase within promyelocytic leukemia nuclear bodies that are involved in transcription of IFN-induced genes [35]. Accordingly, RHA may not only be induced by IFN, but could also regulate its downstream effects. Therefore appropriation of RHA by viruses during their replication would not only boost viral transcripts, but may also blunt the innate immune response.

Our observations of interplay between RHA and PKR strengthen the perspective that helicases are key signaling molecules. Helicases had been thought of as terminal proteins in signal cascades that elicit appropriate responses by remodeling RNA and DNA. The data here underpin previous findings with RIG-I and IFIH-I to support a primary role for helicases as immediate players in the innate immune response [36]. We demonstrate that just as the CARD domains of these helicases and their associated adaptor molecules mediate signal transduction, the RBM of RHA mediates the association with PKR. Importantly, by identifying an inhibitory effect of phosphorylation on the function of RHA, we present compelling evidence of this association, with resulting effect upon the enzyme's function. Correspondingly, peptides within RHA's RBD, that interacts with PKR, enhance the infectivity of HIV-1. The data support a paradigm by which the function of a class of RNA-responsive proteins are coordinated or exacerbated by interaction via their RBMs. The consequence of this could be considerable, as at least 17 human proteins have been described that encode RBMs. Moreover, gene deletion studies highlight the importance of these proteins. Disruption of the RBM-containing ribonuclease Dicer, TARBP, the adenosine deaminase ADAR-1, and, as discussed, RHA, is embryonically lethal in murine models [32], [37]–[39]. Similarly, mice null for the PKR-activator PACT, spermatid perinuclear RNA-binding protein (STRBP), and the testis-specific mRNA editor TENR, which all encode RBMs, have retarded growth, increased mortality and/or reduced fertility (G.C. Sen, unpublished results; L. Saunders and G.N. Barber, unpublished results; [40]–[42]. PKR has previously been reported to associate with four members of this family of proteins. As mentioned the kinase is activated by PACT, and conversely inhibited by TARBP, in addition to the nuclear factor of activated T-cells, NF90, as well as the antiviral protein ADAR1 [43],[44]. The association here between PKR and RHA via their RBMs strengthens an emerging paradigm whereby this motif acts as a signaling domain to coordinate the dsRNA-response as has been identified for the CARD domains of the cytoplasmic helicases RIG-I and IFIH-I, or the TIR domains of TLRs and their adaptor molecules.

## Materials and Methods

### Plasmids and Reagents

Full-length RHA and truncated GST-RHA fusion plasmids were constructed as described by Nakajima et al. [34]. RHA was expressed for protein purification as a recombinant baculovirus as described by Lee et al. [30]. Wild-type PKR was expressed as described previously by Gabel et al. [16]. Truncated RHA constructs, encoding the N-terminal 262 amino acid (pRHARBD), the first 76 amino acids (pRHARBM1), or residues 169 to 262 (pRHARBM2), were generated in pCMVFlag (Sigma). Other plasmid constructs were gifted by others as listed in the acknowledgments.

Gene silencing was achieved though RNA interference using the chemically synthesized siRNAs, AAAUUUUCUGUAUGCCUGG, CAGCCAAAUUAGCUGUUGA, AATGTTCTTCTGGAAGTCCAG, and GCUGACCCUGAAGUUCAUCUU, targeting *rha*, *pkr*, *lamin A/C*, and *egfp* transcripts (Dharmacon). All other reagents were purchased from Sigma unless otherwise indicated.

### Cell Culture and Treatments

Adherent cells were maintained in DMEM, while suspension cells were cultured in RPMI supplemented with 10% fetal bovine serum and cells were grown at 37°C with a humidified 95% air, 5% CO_2_ atmosphere. Murine (C57/BL6) PKR null MEFs were transformed with the pBeloBAC construct encoding an approximately 60 kbp genomic fragment that encompassed the gene and promoter elements of human *pkr* as described previously [45],[46].

Reporter assays were performed in HEK293T cells at 20–60% confluency in 6-well dishes (Falcon). Cells were transfected using the calcium phosphate method with 300 ng pLTR-EGFP, 2 ng pSV2tat72, 10 ng of a control reporter pβactin-RL and 4 nM siRNA per well. Cells were collected 24, 48, and 72 hours after transfection, Assays to measure the effect of the pRHARBD were performed in HEK293T cells cultured in 24 well dishes transfected with 50 ng of pLTR-EGFP, 10 ng pβactin-RL and 0, 10, 20, 40, 80, 160, or 640 ng of either pRHARBD or pCMVFlag DNA. The cells were cultured for 62 hours. HEK293T cells were washed with phosphate-buffered saline (PBS), and lysed in Promega's passive lysis buffer for fluorescence and luciferase measurements using a Wallac Victor3 plate reader (Perkin-Elmer). Fluorescence values were normalized to the total protein level quantified using the Bradford assay (BioRad) and also compared to an internal reporter quantified by *Renilla* luciferase assay (Promega). All experiments were performed in triplicate and independently replicated a minimum of three times.

PKR was activated in MEFs by adding 100 μg/ml pIC to the culture supernatant for 2 hours. Alternatively, THP-1 cells were treated with 10 μg/ml *E. coli* LPS for 2 hours as described by Gusella et al. [47]. Finally, HEK293T transfected with pcDNA-PACT/ΔPACT were temporally treated with Actinomycin-D as described by Peters et al. [20].

### Virus Production and Infection

HIV-1 particles were produced by polyethylenimine transfection of HEK293T cells with 5 μg of pNL4-3-Luc-RE proviral DNA, 2.5 μg of pNLA1, and 2 μg of each RHA construct (pRHARBD, pRHARBM1, or pRHARBM2). Viral particles were harvest after 36 hours, purified from the supernatant and concentrated by ultracentrifugation through 20% sucrose, using ultracentrifuge at 87,000×g for 1 hour at 4°C in a Beckman centrifuge, and virus pellets were eluted in PBS, and quantitated with the HIV-1 Antigen p24-CA MicroELISA Vironostika system (Organon Teknika). Equivalent amounts of virus were used to infect 1×10^6^ MT-2 cells maintained in RF10 (Gibco/BRL), supplemented with 2 mM L-glutamine and 24 μg/ml gentamicin for 2 hours at 37°C. Residual virus was removed by washing with PBS and cells were resuspended in RF10, then cultured at 37°C for 48 hours, before washing with PBS and harvesting in Cell Culture Lysis Reagent (Promega). The success of a single round of infection was determined by the level of luciferase activity, measured by luciferase assay (Promega) using a Fluorostar plate reader (BMG). HIV-1 infectivity was assessed in three independent experiments with two or four replicates at each occasion.

### Reverse Transcriptase Activity Assay

Ten μl of non-concentrated viral supernatant was mixed with 10 μL of 0.3% NP40, followed by addition of 40 μL reverse transcriptase (RT) reaction cocktail containing 5 μg/ml of the template primer poly(rA)-(dT)_15_ (Amersham Pharmacia Biotech), in 50 mM Tris-HCl (pH 7.8), 2 mM DTT, 5 mM MgCl_2_, 7.5 mM KCl, and 0.5 mCi α^33^P-dTTP. Following incubation for 2 hours at 37°C, 8 μL of the reaction mixture was spotted onto DEAE81 ion-exchange paper (Whatman) and washed six times in 300 mM NaCl and 30 mM sodium citrate to remove unincorporated α^33^P-dTTP. RT activity was determined by the level of α^33^P-dTTP using a Wallac 1450 Microbeta-Plus liquid scintillation counter (Perkin-Elmer).

### Immune Analysis

Human PKR was immunoprecipitated using the mouse monoclonal antibody 71/10 [48]. PKR was detected in Western blot with multiple redundant antibodies, including a rabbit monoclonal antibody YE350 from Abcam (for human PKR), and rabbit polyclonal antibodies D20, and B10 from Santa Cruz Biotechnology. Activation of PKR in vivo was confirmed by detecting phosphorylation of eIF2α using a rabbit anti-phospho-eIF2α (Ser51) antibody from Stressgen. GAPDH and GFP were detected in Western blots using mouse monoclonal antibodies from Chemicon and Roche, respectively. Endogenous RHA was detected in Western blot analysis and immunoprecipitated from whole-cell lysates using a rabbit polyclonal antibody [30], a rabbit polyclonal antibody ab26271, and a mouse monoclonal ab54593 from Abcam. Recombinant HA-tagged RHA was immunoprecipitated and detected in Western blots using the monoclonal antibody HA.11 from Covance. Phosphorylated amino acids were detected using rabbit polyclonal anti-phosphoserine, and mouse monoclonal anti-phosphothreonine antibodies from Zymed Laboratories (Invitrogen). Cells were collected in lysis buffer (50 mM Tris-HCL [pH 7.4], 150 mM NaCl, 50 mM NaF, 10 mM β-glycerophosphate, 0.1 mM EDTA, 10% glycerol, 1% Triton X-100, and protease inhibitors). Immune complexes were resuspended in loading buffer (125 mM Tris-HCl [pH 6.8], 4% SDS, 20% glycerol, 10% β-mercaptoethanol, 1% Bromophenol Blue) for separation by SDS-polyacrylamide electrophoresis (SDS-PAGE). Separated proteins were visualized by staining with BioRads Coomassie, or Silver Stain Plus reagents. Stained protein bands were excised from the gel and analyzed by Maldi-ToF. Alternatively, separated proteins were electrophoretically transferred to either Immobilon-P, or Immobilon-FL membrane (Millipore) for immunoblotting using horseradish peroxidase-linked secondary antibody and ECL from Amersham, or fluorescently labeled (680 and 800 nm) secondary antibodies (Invitrogen, Molecular Probes), respectively. Fluorescently labeled antibodies were detected and quantitation using the Odyssey infrared imaging system (Li-Cor). Replicate experiments to quantitate RHA phosphorylated by PKR in vivo recorded mean values of phosphorylated residues on RHA coimmunoprecipitated with PKR of 1.6+/−0.9 for phosphoserine and 5.7+/−1.3 for phosphothreonine. No phosphorylated residues were detected with these phospho-specific antibodies in RHA directly immunoprecipitated. The values of total RHA coimmunoprecipitated with the anti-PKR antibody were 55.3+/−5.8, while that directly immunoprecipitated with the anti-RHA antibody was 111.5+/−13.2 in this experiment.

### Protein Purification and Interaction Assays

GST and His-tagged proteins were purified from *E. coli* and Sf-9 insect cells on either glutatione-Sepharose 4B beads (Amersham) or Ni-NTA agarose (Qiagen) according to the manufacturer's protocols. To map protein interactions, PKR was synthesized in an in vitro coupled transcription–translation system (Promega) with ^35^S-methionine (NEN-DuPont), then incubated in cleared *E. coli* lysate with protease inhibitors with GST-fused RHA constructs for 2 hours at 4°C. Supernatant, containing unbound proteins, was removed after 500×g centrifugation. Recovered beads were rinsed five times with bead-binding buffer (50 mM K_3_PO_4_ [pH 7.5], 150 mM KCl, 1 mM MgCl_2_, 10% glycerol, 1% Triton X-100 and protease inhibitors). The resin-bound proteins were eluted with loading buffer and separated by SDS-PAGE, then visualized by autoradiography.

The experiment on the effect of RNA on the interaction between PKR and the 263 amino acid GSTRHA-fusion peptide was conducted as above with an additional step. Approximately 20 μg of the GSTRHA peptide was incubated with a 16 bp dsRNA at 10, 100, or 1000-fold excess for one hour prior to addition of ^35^S-labeled PKR. The 16 bp dsRNA was synthesized in vitro using T7 RNA polymerase then gel purified from an SDS-PAGE gel.

To measure the relative affinity of unphosphorylated or phosphorylated RHA for RNA, the 263 amino acid N-terminus of RHA was synthesized in vitro with either ^35^S-methionine during the synthesis reaction or γ^32^P-ATP in a PKR kinase assay. Labeled proteins were incubated in binding buffer (20 mM Tris-HCL [pH 7.4], 200 mM NaCl, and 5 mM DTT) with pIC conjugated to agarose beads (Promega) for an hour, then washed with binding buffer five times, eluted with loading buffer and separated by SDS-PAGE. Recovered proteins were detected by exposure to a phosphor screen, imaged with a Storm-840 scanner, and quantified with ImageQuant software (Molecular Dynamics).

### Phosphorylation Analysis

For kinase assays, full-length RHA, truncated GSTRHA fusion proteins, and the PKR substrate B56α [49] were incubated with recombinant PKR in 30 μl DBGA buffer (10 mM Tris-HCl [pH 7.6], 50 mM KCl, 2 mM [CH_3_COO]_2_Mg4H_2_O, 7 mM β-mercaptoethanol, 20% glycerol), 20 μl of DBGB buffer (2.5 mM MnCl_2_ in DBGA), 5 μl of ATP mixture (10 μM ATP and 1.5 μCi of γ^32^P-ATP/ml), and 5 μl of pIC (12 ng/μl) at 30°C for 10 minutes. Phosphorylated proteins were denatured in loading buffer and separated by SDS-PAGE, then visualized by autoradiography. The levels of total proteins in the SDS-PAGE gel were visualized by staining with Coomassie blue. In vivo phosphorylation of RHA was detected as described above (Immune analysis).

### Mass Spectrophotometry

Protein were excised from SDS-PAGE gels and washed in 50% ethanol, 5% acetic acid, reduced and alkylated with DTT and iodoacetamide. The gel slices were dehydrated in acetonitrile and dried in a speed-vac, then digested in 20 ng/ml Trypsin in 50 nM ammonium bicarbonate overnight at room temperature. Released peptides were extracted from the polyacrylamide with 50% acetonitrile with 5% formic acid. The extract was evaporated for LC-MS analysis using a Finnigan LTQ linear ion trap mass spectrometer. Two μl volumes of the extract were injected and the peptides eluted from the column by acetonitrile in a 50 mM acetic acid gradient at a flow rate of 0.2 μl/minute. The microelectrospray ion source was operated at 2.5 kV. Samples were also analyzed by Maldi-ToF. Data collected in the experiment was used to search the NCBI non-redundant database with the search program TurboSequest.
